# Hospital-Based Clinical Profile and Management Patterns of Keratoconus in Riyadh City, Saudi Arabia: A Multi-Center Cross-Sectional Study

**DOI:** 10.3390/medicina62010122

**Published:** 2026-01-07

**Authors:** Khaled Alzahrani, Ali Alrashah, Abdullah Almaznai, Hamad Alzamil, Fatimah Alhamad, Munirah Alonazi, Hanan Alqahtani, Hadeel Alamer, Nourah Alfaifi, Shariefah ALmalki, Khaled Alrashah, Jawaher Alshehri, Seham Eldeeb

**Affiliations:** 1 Optometry Department, King Fahad Armed Forces Hospital, Jeddah 23311, Saudi Arabia; khaledod@gmail.com; 2 Research Administration, Najran Health Cluster, Najran 66252, Saudi Arabia; alalrashah@moh.gov.sa (A.A.); njalfaify@moh.gov.sa (N.A.); 3 Optometry Department, King Saud Medical City, Riyadh 11461, Saudi Arabia; aalmaznai@ksmc.med.sa (A.A.); ab-ya2@hotmail.com (S.A.); 4 Ophthalmology Department, Security Forces Hospital, Riyadh 11481, Saudi Arabia; ha.alzamil@hotmail.com; 5 Binrushd Ophthalmic Centre, Riyadh 12311, Saudi Arabia; fatimah.h.ksu@gmail.com; 6 Royal Commission of Jubail, Jubail Industrial City 35718, Saudi Arabia; dr.munirahalonazi@gmail.com; 7 College of Applied Medical Sciences, King Saud University, Riyadh 11451, Saudi Arabia; halqahtani433@gmail.com; 8 Optometry Division, King Abdullah Specialized Children’s Hospital, Riyadh 11426, Saudi Arabia; alamerha1@mngha.med.sa; 9 Comprehensive Specialized Clinic for Security Forces, Najran 12564, Saudi Arabia; khaled_n777@hotmail.com; 10 Optometry Department, Faculty of Applied Medical Sciences, Al-Baha University, Al-Baha 65431, Saudi Arabia; j.alshehri@bu.edu.sa; 11 Faculty of Medicine, Zagazig University, Zagazig 44519, Egypt

**Keywords:** keratoconus, corneal ectasia, corneal cross-linking, Pentacam

## Abstract

*Background and Objectives*: Keratoconus (KC) is a progressive ectatic corneal disease that can cause irregular astigmatism and visual impairment. To describe the demographic and clinical profile of KC patients attending major eye care centers in Riyadh City, Saudi Arabia, and to explore associations with laterality, disease severity, and management patterns. *Materials and Methods*: This multi-center hospital-based cross-sectional study enrolled consecutive patients with a confirmed diagnosis of KC (new or follow-up) presenting between April 2022 and April 2023. All participants underwent standardized ophthalmic assessment and Scheimpflug tomography (Pentacam). Disease severity was categorized as early, moderate, or advanced using Pentacam-derived keratoconus staging, and ocular parameters (refraction, keratometry, pachymetry, and higher-order aberrations) were compared across severity categories. *Results*: A total of 157 patients (264 eyes) were included (mean age 31.8 years; 56.7% female), with bilateral KC in 68.2%. Eye rubbing (67.8%) and allergic symptoms (61.7%) were common. Keratometric indices and higher-order aberrations differed significantly by severity grade (*p* < 0.001). Management patterns differed by sex and laterality, with corneal cross-linking and glasses reported more frequently in males, and soft contact lens use concentrated among bilateral cases. *Conclusions*: In this hospital-based Riyadh sample, KC was often associated with eye rubbing and allergic symptoms and showed clear stage-dependent worsening of tomographic indices and optical quality. These findings support early detection and targeted counseling on modifiable behaviors, while population-based studies with non-diseased comparators are needed to quantify incidence and prevalence in Riyadh.

## 1. Introduction

Keratoconus (KC) is an eye condition characterized by a gradual thinning and protrusion of the cornea, leading to a conical and irregular shape [1]. Early in the disease process, thinning of the corneal stroma leads to deteriorated and hazy vision, often uncorrectable with prescription glasses [2]. KC typically affects both eyes asymmetrically, with changes primarily in the central or paracentral two-thirds of the cornea [1,2,3]. This condition usually begins in early adolescence and progresses through the mid-30s to 40s [4].

However, the global prevalence and incidence of KC have not been precisely determined. Estimates suggest that KC affects approximately 1 in 400 to 1 in 2000 individuals worldwide. One study reported incidence rates ranging from 5 to 23 per 10,000 individuals and a prevalence of 5.4 per 10,000 [5].

In a recent meta-analysis by Mohamed (2025), the pooled prevalence of keratoconus was 3.96% (95% CI: 3.75–4.16) in the Eastern Mediterranean region [6]. Comparative studies indicate that Asians are more susceptible to KC than Caucasians, with incidence rates of 229 and 57 per 100,000, respectively, suggesting a relative prevalence ratio of 4 to 1 [7,8,9]. Geographical location is recognized as a significant determinant of KC incidence rates [10].

Riyadh is the capital and largest city in the central region of Saudi Arabia, hosting a rapidly growing and diverse population. Despite increasing use of corneal tomography and expanding access to corneal cross-linking, local data describing how KC presents and is managed in routine clinical practice in Riyadh remain limited. Therefore, this study aimed to (i) describe the demographic profile and clinical presentation of KC patients attending three major eye care centers in Riyadh; (ii) examine associations between laterality (unilateral vs. bilateral KC) and patient-level characteristics; (iii) compare key tomographic and optical quality parameters across KC severity grades; and (iv) describe management patterns in this hospital-based sample.

## 2. Materials and Methods

This multi-center, hospital-based cross-sectional study was conducted in Riyadh City over 12 months (April 2022 to April 2023) in three ophthalmology/optometry settings (King Saud Medical City, Ibn Rushd Eye Centre, and Security Forces Hospital).

Consecutive patients with a clinical diagnosis of keratoconus attending routine outpatient visits during the study period were invited to participate, including both newly diagnosed and previously diagnosed cases (follow-up). Participants younger than 9 years, those with secondary corneal ectasia (e.g., post-refractive surgery), other primary corneal diseases (e.g., infectious keratitis, dystrophies), major ocular trauma, or inability to provide consent were excluded.

Standardized data collection included socio-demographic characteristics (age, sex, education, and residency), relevant medical history (family history/consanguinity, allergy symptoms, and eye rubbing), and laterality (unilateral/bilateral). Clinical evaluation included uncorrected and best-corrected visual acuity, refraction, slit lamp findings (e.g., corneal thinning, scarring, Vogt’s striae, Fleischer’s ring, Munson’s sign), and intraocular pressure.

Corneal imaging was performed using the OCULUS Pentacam^®^ (OCULUS Optikgeräte GmbH, Wetzlar, Germany) to obtain anterior and posterior keratometry, pachymetry (including thinnest corneal thickness), and corneal higher-order aberrations (HOA). KC diagnosis was confirmed based on compatible clinical findings and tomographic evidence of corneal ectasia. Disease severity was graded using Pentacam-derived keratoconus staging (Topometric/KC Staging display) and categorized as early, moderate, or advanced for analysis [11].

The study was conducted in accordance with the guidelines set forth by the Institutional Committee for the Protection of Human Subjects, which were adopted by the 18th World Medical Assembly in Helsinki, Finland, with subsequent modifications. Ethical approval was obtained from the Institutional Review Board of King Saud Medical City, Riyadh, Saudi Arabia (protocol code H1R1-30-Jun22-01; date of approval: 24 July 2022), and by the Research Ethics Committee of Security Forces Hospital, Riyadh, Saudi Arabia (approval no. 22-589-25; date of approval: 17 April 2022). Written informed consent was taken from all participants in the study. Written informed consent was secured from all participants or guardians. Data were anonymized and stored securely.

Statistical analyses were performed using SPSS version 24 (IBM Corp., Armonk, NY, USA). Categorical variables were summarized as frequencies/percentages and compared using chi-square or Fisher’s exact tests. Continuous variables were summarized as mean ± SD or median (IQR) as appropriate and compared using the Mann–Whitney U test (two groups) or the Kruskal–Wallis test (three severity grades), with post hoc pairwise testing where applicable. Patient-level analyses were used for socio-demographic characteristics and laterality (Tables 1 and 3). Eye-level analyses were used for corneal parameters and HOA by severity grade (Tables 4 and 5). Inter-hospital comparisons of patient characteristics were performed as reported in the Results Section. In all tests, a *p*-value ≤ 0.05 was considered statistically significant.

## 3. Results

Regarding the socio-demographic characteristics of the study participants, “Table 1”, the mean age was 31.8, and 56.7% were female. Almost half of the study sample was collected from Ibn Rushd Eye Centre. Most of the study participants were Saudi citizens from the central region of Saudi Arabia. Only 8.3% of the study participants reported a positive family history of keratoconus. Almost half of the study sample had positive consanguinity. Bilateral keratoconus was found in 68.25% of the participants.

**Table 1:** Sociodemographic characteristics of the participants studied.

Variables	Study Participants (n = 157)
Age (years):	
Mean ± SD	31.8 ± 7.3
Range	16.0–53.0
Gender:	
Males	68 (43.3%)
Females	89 (56.7%)
Hospital:	
Ibn Rushd Eye Centre	75 (47.8%)
Security Forces Hospital	48 (30.6%)
King Saud Medical City	34 (21.7%)
Nationality:	
Saudi	145 (92.4%)
Non-Saudi	12 (7.6%)
Residence:	
Central region	152 (96.8%)
Other regions	5 (3.2%)
Family history:	
Positive	13 (8.3%)
Negative	144 (91.7%)
Consanguinity:	
Positive	85 (54.1%)
Negative	72 (45.9%)
Laterality:	
Bilateral	107 (68.2%)
Unilateral	50 (31.8%)

The study sample comprised 264 affected eyes, “Table 2”. The most common clinical features were eye rubbing (67.8%), Vogt’s striae (49.2%), monocular diplopia (24.6%), and Fleischer’s ring (21.6%). Refractive surgery and eye trauma were reported in 2.3% and 0.4%, respectively. The distribution of keratoconus stages was as follows: early (39.8%), moderate (36.4%), and advanced (23.8%). The most frequently reported treatments were hard contact lens (47.7%), CXL combined with other therapies (24.6%), and CXL alone (12.1%). Less frequently reported treatments included soft contact lenses (6.8%), PKP (3.4%), glasses (2.7%), and ICRS (2.7%).

There were statistically significant associations between keratoconus laterality and the socio-demographic characteristics of the study participants, “Table 3”. Unilateral keratoconus was more associated with older age than bilateral cases (33.8 ± 6.8 versus 30.8 ± 7.3, respectively). Security Forces Hospital reported more unilateral cases (50.7%) compared to the other hospitals involved: Ibn Rushd Eye Centre (11.8%) and King Saud Medical City (16.7%). In addition, positive consanguinity was significantly associated with bilateral keratoconus (*p* < 0.001).

**Table 3:** Association between laterality and socio-demographic characteristics of the study participants.

Variables	Keratoconus Patients		Test of Sig.	*p*-Value
	Bilateral	Unilateral		
Age (years):			T	
Mean ± SD	30.8 ± 7.3	33.8 ± 6.8	2.4	0.01 *
Range	16.0–46.0	23.0–53.0		
Gender:			χ^2^	
Males (n = 68)	46 (67.6%)	22 (32.4%)	0.01	0.9
Females (n = 89)	61 (68.5%)	28 (31.5%)		
Hospital:			χ^2^	
Ibn Rushd Eye Centre (n = 75)	30 (88.2%)	4 (11.8%)	23.7	<0.001 *
Security Forces Hospital (n = 48)	37 (49.3%)	38 (50.7%)		
King Saud Medical City (n = 34)	40 (83.3%)	8 (16.7%)		
Nationality:			χ^2^	
Saudi (n = 145)	96 (66.2%)	49 (33.8%)	3.3	0.07
Non-Saudi (n = 12)	11 (91.7%)	1 (8.3%)		
Residence:				
Central region (n = 152)	103 (67.8%)	49 (32.2%)	Fisher	0.9
Other areas (n = 5)	4 (80.0%)	1 (20.0%)		
Family history:			χ^2^	
Positive (n = 13)	11 (84.6%)	2 (15.4%)	1.8	0.2
Negative (n = 144)	96 (66.7%)	48 (33.3%)		
Consanguinity:			χ^2^	
Positive (n = 85)	68 (80.0%)	17 (20.0%)	12.0	<0.001 *
Negative (n = 72)	39 (54.2%)	33 (45.8%)		

* Statistically significant.

**Figure 1:**
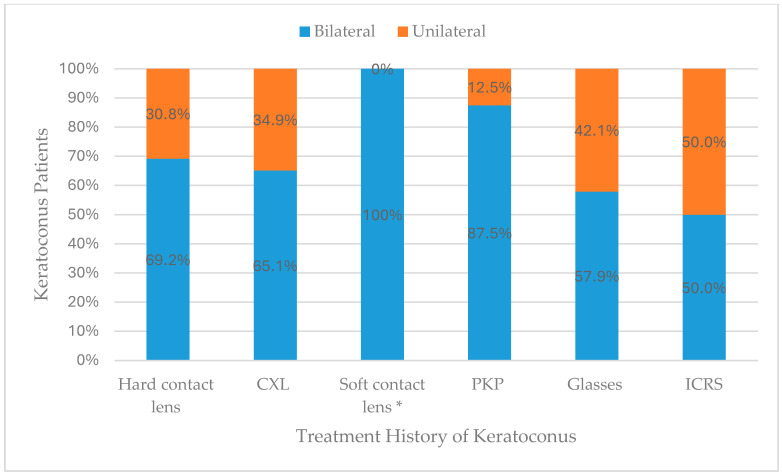
Comparison between unilateral and bilateral keratoconus patients in treatment history. * Statistically significant.

**Figure 2:**
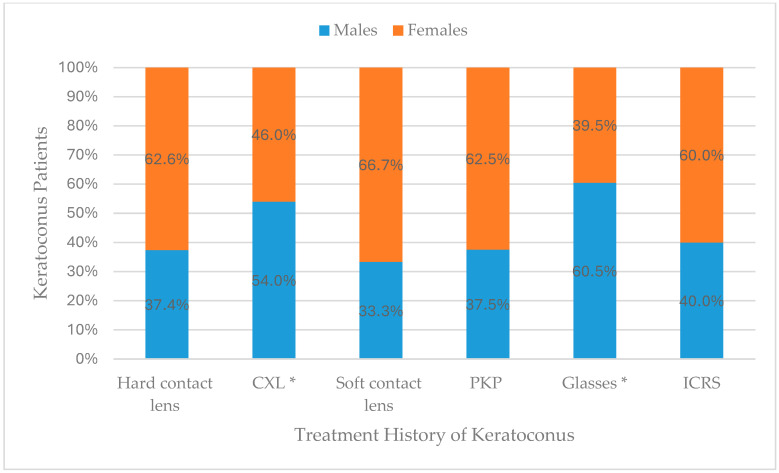
Comparison between male and female keratoconus patients in treatment history. * Statistically significant.

When comparing unilateral and bilateral keratoconus patients in treatment history, there was a statistically significant difference, “Figure 1”. Soft contact lenses were reported in bilateral keratoconus only. None of the unilateral cases reported using soft contact lenses. In addition, when comparing male and female keratoconus patients in treatment history, there were statistically significant differences, “Figure 2”. CXL and glasses were significantly more reported by males (54.0% and 60.5%, respectively).

Pentacam keratoconus indices in different stages of the affected eyes are presented in “Table 4”. Regarding the corneal curvature parameters (SimK1, SimK2, Average K), a consistent increase in keratometry readings reflects the progressive steepening of the cornea as keratoconus advances (*p* < 0.001). Corneal thickness (pachymetry) significantly decreases from early to advanced stages, a hallmark feature of keratoconus pathophysiology. In addition, aberrations (HOA and RMS) increase substantially with disease severity (*p* < 0.001).

**Table 4:** Pentacam keratoconus indices in different stages of the affected eyes.

Variables	Keratoconus Stage			KW	*p*-Value
	Early	Moderate	Advanced		
SimK1 (Diopters):					
Median	43.5	46.1	55.1	101.8	<0.001 *
IQ-Range	42.2–44.8	43.5–48.5	48.8–60.7		
SimK2 (Diopters):					
Median	46.6	50.8	59.5	138.4	<0.001 *
IQ-Range	45.0–48.2	48.8–53.4	53.9–65.0		
Average K (Diopters):					
Median	45.0	48.3	57.3	122.0	<0.001 *
IQ-Range	43.8–46.5	46.1–50.5	50.4–62.3		
Pachymetry (µm):					
Median	478.5	438.5	396.0	64.6	<0.001 *
IQ-Range	447.5–510.3	409.3–464.8	363.0–448.3		
HOA (µm):					
Median	1.62	2.63	3.69	80.6	<0.001 *
IQ-Range	1.15–2.2	2.04–3.38	2.6–5.15		
RMS (µm):					
Median	5.43	8.82	14.67	58.5	<0.001 *
IQ-Range	1.48–7.73	3.58–13.77	7.68–17.56		

* Statistically significant.

There were statistically significant differences between keratoconus stages in the refraction variables, “Table 5”. Statistically significant differences were observed in both sphere and cylinder values across the early, moderate, and advanced stages (*p* < 0.001). At the same time, changes in axis and IOP were not statistically significant (*p* = 0.2 and *p* = 0.5, respectively).

**Table 5:** Refraction variables and intraocular pressure in different keratoconus stages of the affected eyes.

Variables	Keratoconus Stage			KW	*p*-Value
	Early	Moderate	Advanced		
Sphere (Diopters):					
Median	−1.5	−2.5	−3.5	22.9	<0.001 *
IQ-Range	−2.8–0.0	−3.8–−0.5	−6.6–−1.8		
Cylinder (Diopters):					
Median	−3.3	−5.0	−6.0	62.3	<0.001 *
IQ-Range	−4.5–−2.3	−6.0–−3.8	−6.9–−4.7		
Axis (Degrees):					
Median	75.0	110.0	110.0	3.1	0.2
IQ-Range	30.0–130.0	45.0–140.0	30.0–165.0		
IOP (mmHg):					
Median	13.0	13.0	13.0	1.3	0.5
IQ-Range	12.0–14.0	12.0–14.0	10.4–14.0		

* Statistically significant.

## 4. Discussion

In this Riyadh hospital-based cross-sectional study, one specialty hospital and two general hospitals were involved. Ibn Rushd Eye Centre, the specialty hospital involved in the study, contributed 47.8% of the sample studied. The age distribution aligns with Alghamdi et al. (2024) [12]. They reported a mean age of 30–31 years for patients with keratoconus/ectasia in multiple medical centers in Saudi Arabia, with a predominance of males (59.1%) among 215 patients; bilaterality was 98.6% (diagnosis-based) in that dataset.

Earlier phenotyping at King Khaled Eye Specialist Hospital also described male predominance (2:1) and presentation in the second to third decades among national referrals to Riyadh’s tertiary center [13]. Tertiary referral studies from Riyadh often pool patients from multiple regions, resulting in different geographic mixes and severity spectra. This referral dynamic partly explains disparities in age at diagnosis, sex ratio, and bilaterality. This explains the low bilateral rate (68.2%) in our study. In addition, specialty centers receive progressed/confirmed bilateral cases, whereas hospital-based samples capture a broader spectrum, including earlier or unilateral presentations. This gradient (hospital vs. tertiary) is a recurring theme in Saudi datasets and should be acknowledged when comparing bilaterality across settings.

The family history prevalence (8.3%) in our study is lower compared to several local and regional reports. In Najran and eastern provinces, the family history of keratoconus was 48% and 21%, respectively. The variation is possibly due to under-ascertainment or differences in case definitions and data collection methods [14,15]. By contrast, consanguinity (54.1%) in our sample closely mirrors national background rates and is consistent with Saudi keratoconus cohorts, which frequently report endogamy/consanguinity (50–55%), supporting a genetic contribution to disease risk in this population [16,17]. Beyond descriptive alignment, multiple studies (regional and international) have linked parental consanguinity and positive family history to increased KC risk [18,19].

The predominance of behavioral (eye rubbing) and structural (Vogt’s striae, Fleischer’s ring) KC indicators reaffirms the value of routine slit lamp and tomography screening in primary and secondary care. Similar proportions have been documented in other Saudi cohorts, ranging from 69% in the Najran cluster to 65–72% in studies from the Eastern Province, underscoring its role as a modifiable behavioral risk factor [14,15]. Vogt’s striae and Fleischer’s ring remain the most characteristic slit lamp findings, reported in 40–60% of keratoconus eyes and nearly identical to the 49% and 22% frequencies observed in our study [20]. Monocular diplopia was observed in roughly one-quarter of eyes, comparable to the study of Alghamdi et al. (2024) [12], and reflects optical irregularity from asymmetric corneal steepening [21]. The low proportion of post-refractive surgery and trauma suggests that iatrogenic or traumatic ectasia contributes minimally to keratoconus epidemiology in our study population, unlike the higher rates seen in tertiary corneal surgery centers [22].

The stage distribution indicates that most patients presented before severe disease. This pattern is similar to hospital-based data from the Eastern Province (early 37%, moderate 34%, advanced 29%) and contrasts with tertiary referral studies, such as that of King Khaled Eye Specialist Hospital, where advanced disease exceeds 50% due to referral bias [13,15]. The relatively high proportion of early cases in the current sample highlights the increasing public and primary care awareness of keratoconus, as well as the growing adoption of screening tomography (e.g., Pentacam) in Riyadh clinics.

Management trends in our study mirror evolving Saudi practice and greater adoption of conservative and biomechanical stabilizing therapies. Nearly half of the eyes were managed with rigid gas-permeable lenses, aligning with central and western region data showing 45–55% lens use [13,15]. Corneal cross-linking (CXL) is now a cornerstone therapy in Saudi Arabia [23]. The low prevalence of PKP (3.4%) and ICRS (2.7%) contrasts sharply with older national data [24], reflecting a national shift toward earlier CXL intervention and the declining need for transplantation. Limited spectacle use (2.7%) further underscores the inadequacy of regular correction in ectatic corneas, a finding consistent with global keratoconus management patterns [25].

Unilateral keratoconus was associated with older age compared to bilateral disease, suggesting that unilateral presentation may represent either a milder or a later-onset phenotype that progresses more slowly or remains asymmetric for an extended period. Similar age differences between unilateral and bilateral cases have been observed in regional cohorts, including a Najran population study [14], supporting the possibility that age-related biomechanical remodeling and environmental triggers (e.g., chronic eye rubbing or UV exposure) may contribute to asymmetrical disease expression [25].

A significant difference in laterality distribution among hospitals was also evident: Security Forces Hospital reported the highest proportion of unilateral cases (50.7%), compared with Ibn Rushd Eye Centre (11.8%) and King Saud Medical City (16.7%). This disparity likely reflects institutional referral patterns and case cmix differences. The Security Forces Hospital primarily serves working-age adults undergoing occupational screening. In contrast, Ibn Rushd and King Saud centers function as tertiary referral hospitals, receiving patients with more advanced, bilateral, or progressive keratoconus. Comparable institutional variations have been reported by Alabdelmoneam (2012) at King Khaled Eye Specialist Hospital, where more than 90% of cases were bilateral, consistent with the referral of complex diseases [26]. Thus, laterality distribution may be shaped as much by service structure as by biological factors.

Positive consanguinity was strongly associated with bilateral keratoconus (*p* < 0.001). This finding underscores the genetic contribution to disease expression and is consistent with Saudi and Middle Eastern studies reporting higher rates of bilaterality and severity in offspring of consanguineous unions [27,28].

Treatment history among keratoconus patients revealed that soft contact lenses were reported exclusively among bilateral keratoconus cases. This finding suggests that bilateral involvement tends to be associated with more advanced or symptomatic visual distortion, prompting patients to seek earlier optical correction. The complete absence of soft lens use in unilateral cases may also reflect minimal refractive irregularity or adequate visual compensation by the unaffected eye, delaying clinical presentation and treatment initiation.

Comparable patterns have been reported in Saudi and regional studies [14,28]. Internationally, similar findings have been reported in tertiary cohorts from Spain and Brazil, where soft lenses are rarely prescribed for early or unilateral cases [29,30]. Instead, rigid gas-permeable (RGP) and scleral lenses remain the preferred modalities for bilateral disease due to their superior ability to neutralize higher-order aberrations [25]. The selective use of soft lenses in bilateral keratoconus, therefore, reflects a stage-specific management approach consistent with global clinical practice.

In addition, corneal cross-linking (CXL) and spectacle use were reported significantly more often among male patients (54.0% and 60.5%, respectively). These differences may reflect gender-related variation in disease severity at presentation, clinical referral pathways, and health-seeking behavior within the Saudi population. Similar gender-based treatment disparities have been observed in other Saudi and regional studies [23,26]. They reported that males were more frequently managed with CXL and surgical interventions, whereas females tended to rely on optical correction such as contact lenses or glasses.

Internationally, studies from Spain and Turkey have also demonstrated higher CXL utilization in males, correlating with steeper corneal topography and faster progression at baseline [30,31,32]. Conversely, females often exhibit slower progression, possibly due to hormonal or biomechanical factors influencing corneal collagen cross-linking [21,25]. The greater frequency of glasses use among males in the current study may also indicate earlier detection and management of milder cases, in which spectacles can still provide functional correction before significant irregular astigmatism develops.

Regarding corneal curvature parameters, a consistent and statistically significant increase in keratometric readings was observed across the early, moderate, and advanced stages of the disease. This pattern reflects the progressive steepening of the cornea that characterizes the ectatic process of keratoconus. Similar findings were reported by Hashemi et al. (2015) in an Iranian population and by Al-Mahrouqi et al. (2018) in an Omani population, both confirming that curvature parameters increase proportionally with disease progression [27,28].

Corneal thickness demonstrated a significant reduction from early to advanced stages, which is a well-established hallmark of keratoconus pathophysiology and a key diagnostic indicator in tomographic staging [32]. Likewise, higher-order aberrations (HOA) and root mean square (RMS) values increased substantially with disease severity, reflecting an increase in optical irregularity and deterioration in visual quality. These results align with studies by Alió and Shabayek (2006) and Piñero et al. (2012), who demonstrated similar correlations between advanced keratoconus and increased ocular aberrations [21,33].

The current study found statistically significant differences between disease stages in both sphere and cylinder values. The progressive shift toward greater myopia and higher astigmatism with advancing stages mirrors the optical consequences of corneal steepening and irregularity observed tomographically [34]. These patterns are consistent with Saudi studies and international cohorts from Spain and Turkey, where myopic and astigmatic refractive errors increased in proportion to the severity of keratoconus [12,17,21].

In contrast, axis and intraocular pressure (IOP) values showed no statistically significant differences. The variability in astigmatic axis likely reflects asymmetrical cone morphology and the non-orthogonal distribution of corneal steepening, as previously described by Belin and Duncan (2019) [32]. The stability of IOP across disease stages is also in agreement with previous findings that applanation tonometry underestimates true IOP in keratoconus eyes due to reduced corneal rigidity rather than actual hypotony [35].

Strengths and limitations: This study presents contemporary, multi-center, real-world data on the presentation and management of keratoconus in Riyadh, utilizing standardized clinical examinations and Scheimpflug tomography. However, as a hospital-based cross-sectional study, the findings reflect the incidence and prevalence among patients attending the clinic. They cannot be used to estimate the population incidence or prevalence. The absence of an age- and sex-matched non-keratoconus control group prevents direct comparisons of clinical features and risk factors between individuals with keratoconus and those without keratoconus; therefore, associations with allergy and eye rubbing should be interpreted descriptively. Additionally, eye-level analyses may be influenced by within-patient correlation in bilateral cases; this limitation is acknowledged when interpreting stage-based comparisons.

## 5. Conclusions

This multi-center, hospital-based, cross-sectional study provides an updated clinical profile of keratoconus patients attending major eye care centers in Riyadh City. Keratoconus was predominantly bilateral and frequently accompanied by eye rubbing and allergic symptoms. Severity grades were associated with marked deterioration in keratometry, pachymetry, and higher-order aberrations. These findings reinforce the value of early tomography-based detection, structured patient counseling on modifiable behaviors, and timely referral for interventions such as corneal cross-linking. Future population-based studies incorporating appropriate non-diseased comparators and denominators are needed to quantify incidence and prevalence, as well as to clarify local risk determinants in Riyadh.

## Figures and Tables

**Table 2:** Clinical characteristics of the affected eyes.

Variables	Affected Eyes (n = 264)	
	No.	%
Eye rubbing	179	67.8
Vogt’s striae	130	49.2
Monocular diplopia	65	24.6
Fleischer’s ring	57	21.6
Refractive surgery (e.g., LASIK/PRK)	6	2.3
Eye trauma	1	0.4
Keratoconus stage:		
Early	105	39.8
Moderate	96	36.4
Advanced	63	23.8
Treatment history:		
Hard contact lens	126	47.7
CXL and other treatments *	65	24.6
CXL alone	32	12.1
Soft contact lens	18	6.8
PKP	9	3.4
Glasses	7	2.7
ICRS	7	2.7

* Other treatments combined with CXL include glasses, hard contact lenses, or ICRS.

## Data Availability

The data are unavailable due to privacy and ethical restrictions.

## Abbreviations

The following abbreviations are used in this manuscript:

KC	Keratoconus
CXL	Corneal Cross-Linking
PKP	Penetrating Keratoplasty
ICRS	Intrastromal Corneal Ring Segments
IOP	Intraocular pressure
